# Computational modelling of valvular heart disease: haemodynamic insights and clinical implications

**DOI:** 10.3389/fbioe.2024.1462542

**Published:** 2024-11-12

**Authors:** Michael Šeman, Andrew F. Stephens, David M. Kaye, Shaun D. Gregory, Dion Stub

**Affiliations:** ^1^ School of Public Health and Preventative Medicine, Monash University, Melbourne, VIC, Australia; ^2^ Cardio-Respiratory Engineering and Technology Laboratory, Department of Mechanical and Aerospace Engineering, Monash University, Melbourne, VIC, Australia; ^3^ Department of Cardiology – Alfred Health, Melbourne, VIC, Australia; ^4^ Centre for Biomedical Technologies, Queensland University of Technology, Brisbane, QLD, Australia; ^5^ Cardiology and Therapeutics Division, Baker Heart and Diabetes Institute, Melbourne, VIC, Australia; ^6^ School of Medicine, Monash University, Melbourne, VIC, Australia

**Keywords:** cardiovascular model, mathematical model, lumped parameter, aortic stenosis, mitral regurgitation

## Abstract

An aging population and an increasing incidence of cardiovascular risk factors form the basis for a global rising prevalence of valvular heart disease (VHD). Research to further our understanding of the pathophysiology of VHD is often confined to the clinical setting. However, in recent years, sophisticated computational models of the cardiovascular system have been increasingly used to investigate a variety of VHD states. Computational modelling provides new opportunities to gain insights into pathophysiological processes that may otherwise be difficult, or even impossible, to attain in human or animal studies. Simulations of co-existing cardiac pathologies, such as heart failure, atrial fibrillation, and mixed valvular disease, have unveiled new insights that can inform clinical research and practice. More recently, advancements have been made in using models for making patient-specific diagnostic predictions. This review showcases valuable insights gained from computational studies on VHD and their clinical implications.

## 1 Introduction

Valvular heart disease (VHD) is an is an evolving epidemic of an aging population, affecting more the 10% of individuals over the age of 75 (Nkomo et al., 2006). Despite substantial therapeutic advances, it remains a major cause of morbidity and mortality (d'Arcy et al., 2011). Aging, improved cardiovascular survival, and increasing incidence of risk factors such as hypertension, diabetes, and chronic kidney disease, are key drivers for the rising global prevalence of VHD (Coffey et al., 2021). Clinical outcomes are poor when VHD is complicated by other cardiac pathologies, such as adverse cardiac remodelling leading to heart failure or atrial fibrillation (Baumgartner et al., 2017; Alassar et al., 2013; Samaras et al., 2021). As such, early detection and optimizing treatment strategies for patients is paramount.

Investigating the haemodynamic mechanisms of VHD has traditionally been confined to select clinical or animal studies. Studying valvulopathy in the clinical setting has its inherent challenges, including the limited ability to measure or manipulate variables, the need for invasive cardiac measurements, natural physiological intra-patient variability (for example, heart rate or blood pressure) and interpatient heterogeneity due to biological differences (such as age, height, and weight). Furthermore, changing disease patterns, such as mixed and multivalvular pathology, and co-existing cardiac pathology such as heart failure further complicate diagnostic evaluation and clinical decision-making.

**FIGURE 1:**
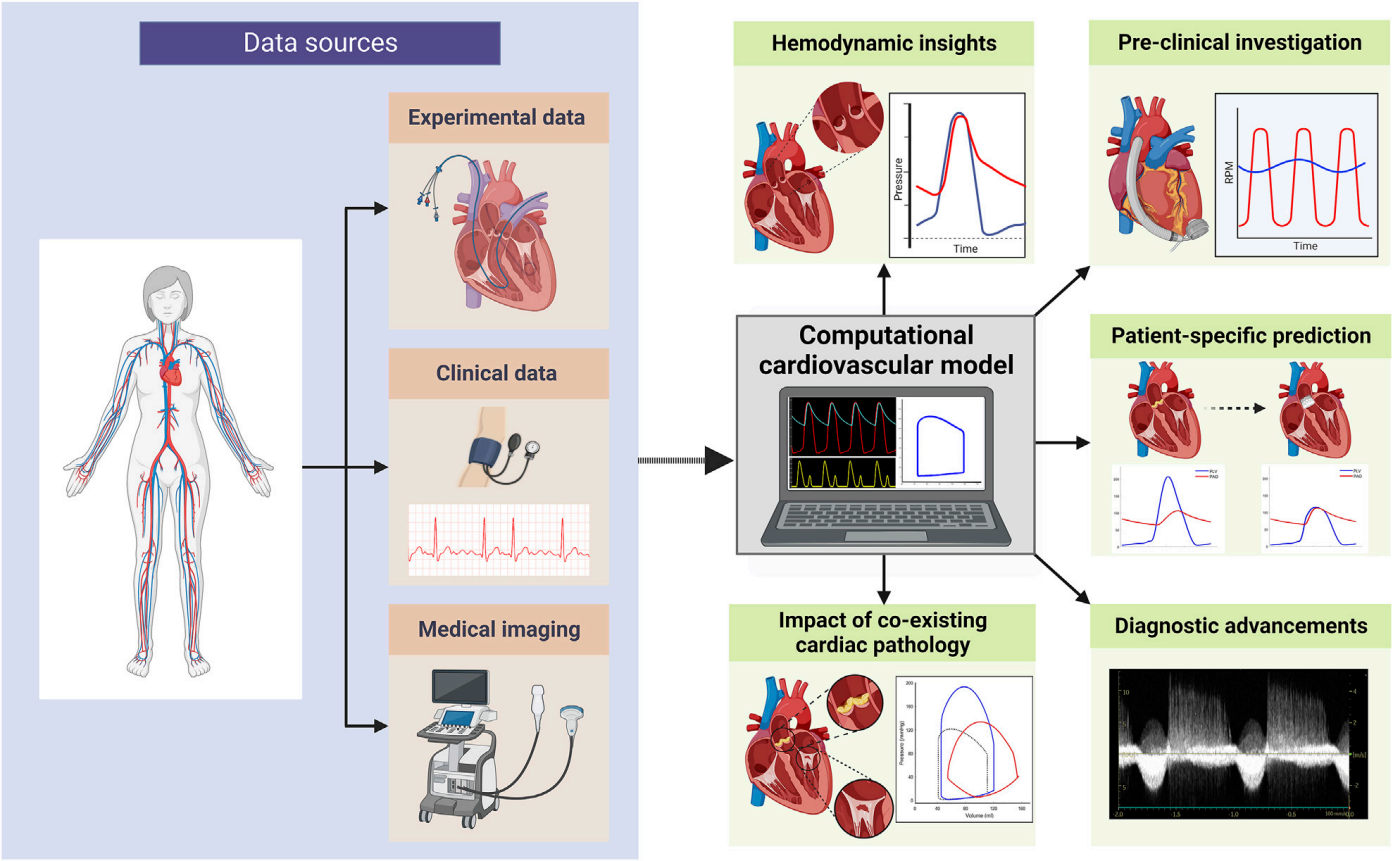
Overview of computational cardiovascular modelling applications in valvular heart disease research (created with BioRender.com).

With the increasing prevalence of VHD and the continuous evolution of treatment options, gaining a deeper understanding of its pathophysiology and natural progression has never been more crucial. For this reason, expanding the armament of research methods to investigate VHD is essential. Computational models of the cardiovascular system are powerful tools to further our understanding of cardiovascular physiology and disease processes and how they may relate to novel therapies (see Figure 1). Such models allow for the creation of detailed virtual representations of cardiovascular structures and functions, which can be manipulated to simulate various physiological states and conditions (Formaggia et al., 2009). This capability provides the means to unveil key mechanistic insights underlying cardiovascular diseases and offers a platform for testing potential therapeutic interventions in a controlled and reproducible manner. Furthermore, computational models overcome the limitations posed by the invasive nature of certain clinical measurements and the inherent variability among patients. By enabling the simulation of a wide range of scenarios and conditions, these models enhance our ability to predict disease progression and treatment outcomes, ultimately informing the development of more effective and targeted therapeutic strategies.

In the simulation of VHD, a comprehensive understanding of the effects of valvular pathology on global cardiovascular haemodynamics within a closed-loop model of the cardiovascular system model can only be achieved through the application of lumped parameter computational modelling (LPM). This method allows for the integration of complex cardiovascular dynamics, including the interactions between the heart chambers, valves, and vasculature. By doing so, it provides a detailed representation of key haemodynamic parameters, including pressure, flow, and volume, within a simplified yet robust framework. This approach offers invaluable insights into the haemodynamic effects of valvular dysfunction, facilitating the prediction of disease progression and assessing potential therapeutic interventions. Furthermore, LPM bridges the gap between clinical observations and theoretical predictions, thereby improving our capacity to diagnose, manage, and treat VHD more effectively.

In this narrative review, we aim to introduce non-experts to the innovative field of computational cardiovascular modelling and its emerging role in VHD research. By elucidating the fundamental principles and showcasing illustrative examples, our goal is to foster a broader appreciation and understanding of computational modelling of VHD. In doing so, we aim to highlight the exciting possibilities and practical applications that computational cardiovascular modelling presents, thereby inspiring further interest and engagement from a diverse audience.

## 2 Concepts in cardiovascular modelling

### 2.1 Mathematical modelling

Mathematical models describe a real-world process in mathematical terms typically through a set of variables and equations. These models are the cornerstone for fields such as physics and engineering, describing natural phenomena such as energy transfer, electromagnetism, and gravity. Physiology, in contrast, is less easily reduced to mathematical equations. This is primarily due to the complexity and variability of biological processes which makes them inherently non-linear, multi-disciplinary, and multi-scaled (Pathmanathan and Gray, 2018). Nevertheless, the use of modelling has led to great advancements in the understanding of cardiovascular physiology and the practice of cardiology. Indeed, many of these are based on well-established physics-based models and principles, having been either directly applied or adapted for use in a biological context. Some notable examples include Hagen–Poiseuille’s law for understanding the relationship between pressure, fluidic resistance, and flow rate, and the Bernoulli principle for describing the pressure drop occurring with increased velocity of blood flow (Formaggia et al., 2009; Hopkins et al., 1991; Sagawa et al., 1973).

**FIGURE 2:**
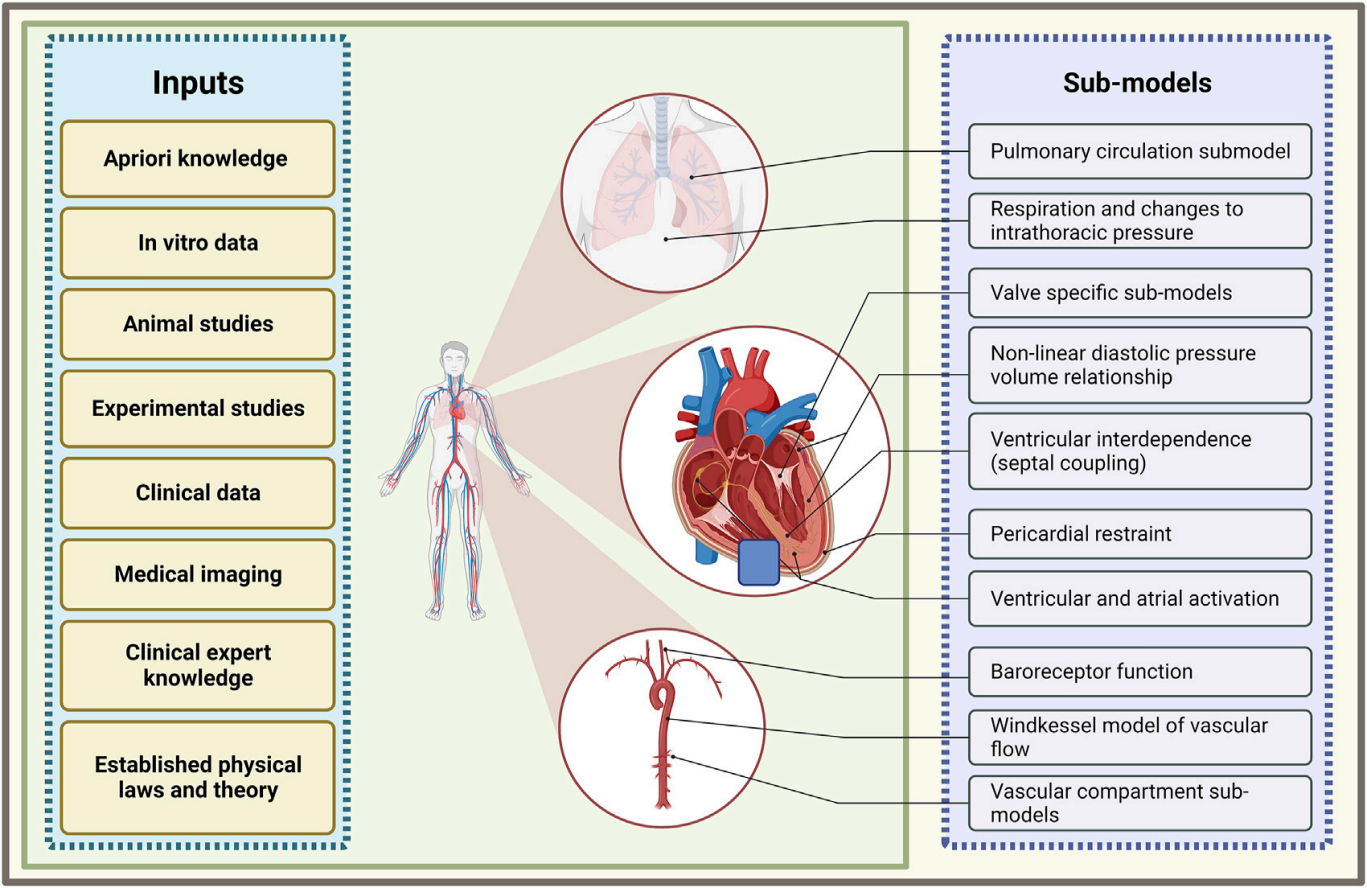
Summary of sub-model components of a closed-loop cardiovascular system model and the inputs which inform their development. Illustration of frequently used models of various cardiovascular processes that are combined to form a multi-compartmental model of a closed-loop cardiovascular system (created with BioRender.com).

Mathematical models of cardiac chamber contractility, transvalvular flow, and flow through vascular compartments have been developed and sequentially integrated to form a closed-loop model of the cardiovascular system (see Figure 2) (Sagawa et al., 1973; Chung et al., 1997; Ursino et al., 1994; Morris et al., 2016). This system-based modelling approach allows for a multitude of physiological and haemodynamic processes, and their interdependencies, to be simultaneously represented. With this approach, the simulation of a variety of disease states is possible. These complex multi-compartment cardiovascular models typically require computer-based methods to execute the model and generate results, such is the reason they are also referred to as computational models.

### 2.2 Reduced-dimensional modelling

In order to effectively model the whole heart and cardiovascular system in unison, each component needs to be simplified with respect to its temporal and spatial representation. The spatial dimensionality of cardiovascular models varies depending on their intended use and application, ranging from zero-dimensional (0D) to three-dimensional (3D) (see Table 1) (Morris et al., 2016). Three-dimensional models, such as those used in computational fluid dynamics, can reveal intricate local fluid flow patterns, such as flow vortices within the left ventricle. Conversely, reduced-dimensional models eliminate spatial dimensionality entirely (0D) or represent one (1D) or two spatial planes (2D). Depending on the model’s purpose, this reduction can translate to decreased computational time without sacrificing accuracy. In reduced-dimensional models, the mathematical representation of cardiovascular components is often lumped together and is known as lumped-parameter modelling - for example, the segments of the aorta may be collectively represented as one whole compartment.

**TABLE 1:** Spatial dimensionality in cardiovascular modelling.

Spatial dimension	Graphical representation	Features	Computational time^a^
0D	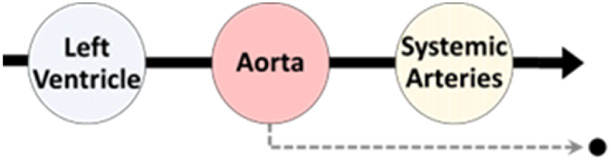	No spatial dimension represented Values for pressure, volume, and directional flow are derived for each modeled section	Seconds
1D	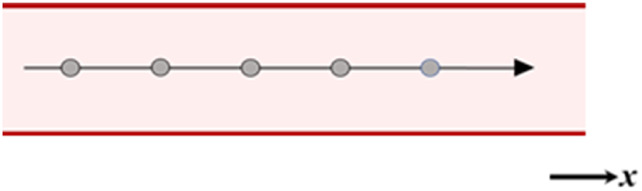	Pressure, volume, and flow can be captured at specified discrete intervals across the length of the vessel Uniform velocity flow profiles are assumed	Seconds to minutes
2D	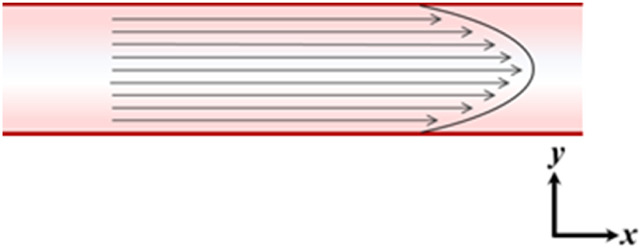	Allows for simulation of velocity field profiles, including parabolic, Womersley, and vortical flow in 2D Simulation localized to one or several connecting vessels or chambers. For closed-loop cardiovascular system modelling, connection to 0D models is required	Minutes to hours
3D	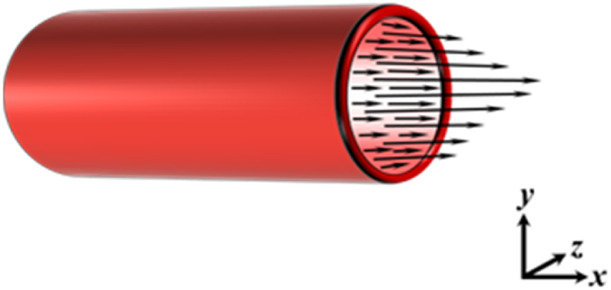	Primary and secondary fluid flow characteristics can be simulated. Capacity for high temporal and spatial resolution Simulation localized to one or several adjoining vessels or chambers. For closed-loop cardiovascular system modelling, connection to 0D models is required	Hours to days

^a^ Typical computational times are presented. Computational times can vary widely depending on the complexity of the model, number and duration of simulations, data collected, software used, type of solver and computer processing power.

**FIGURE 3:**
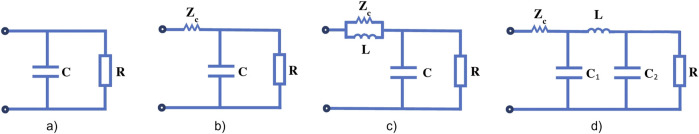
**(A)** Classic two-element Windkessel model; **(B)** three-element Windkessel model; **(C)** four-element Windkessel model and **(D)** five-element Windkessel model. R is peripheral resistance, Z_C_ is characteristic impedance, L is inertance, C is arterial compliance and C_1_ + C_2_ = arterial compliance in the five-element model. (Figure adapted from Zhou et al. (2019)).

The Windkessel model, first described by German Physiologist Otto Frank in 1899 (Frank, 1899), is the most commonly used LPM model to simulate the flow of blood in a vessel or heart chamber. Over the years, improvements to the Windkessel model have led to more accurate representations of physiological pressure and flow waveforms. In the original two-element Windkessel model, resistance and compliance are used simulate flow through a vessel. This evolved to the three-element Windkessel, where an additional resistance value was included to model the impedance imposed by the vessel itself. The effect of inertia to blood flow was accounted for in a four-element model. Finally, the five-element model splits compliance into two components separated by an inertance, to create a more realistic representation of the effect of vessel impedance and compliance (Toy et al., 1985) (see Figure 3).

**FIGURE 4:**
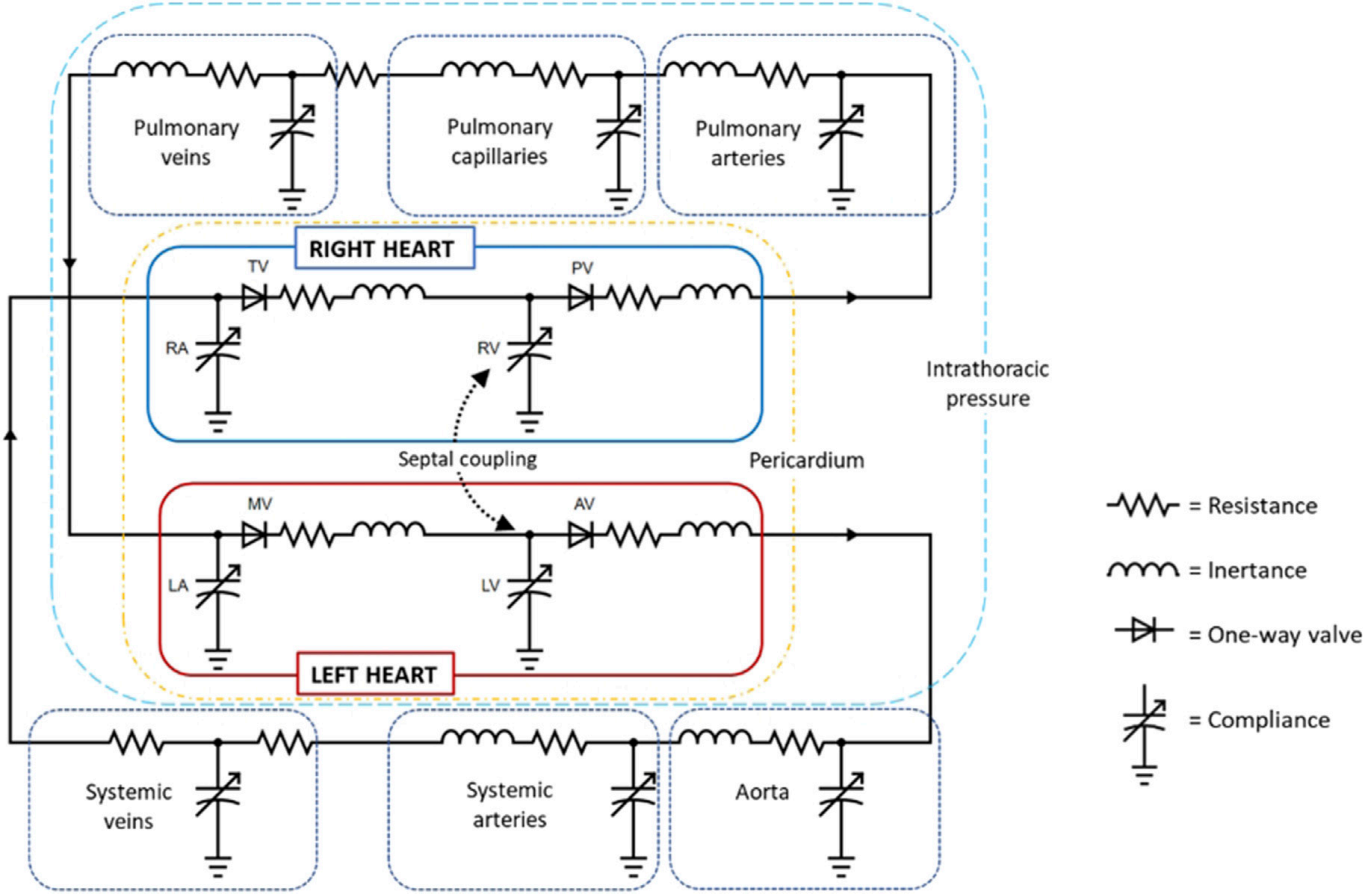
Circuit diagram of a closed-loop cardiovascular system model (created with circuit-diagram.org). Abbreviations: AV, aortic valve; LA, left atrium; LV, left ventricle; MV, mitral valve; PV, pulmonary valve; RA, right atrium; RV, right ventricle; TV, tricuspid valve.

LPMs forming a closed loop model of the cardiovascular system are typically represented by an electrical or hydraulic circuit schematic (Figure 4). The flow of blood is pressure-driven between compartments, where a compartment comprises of elements of resistance, compliance and inertance to blood flow. Flow in and out of the compartments are a function of parameters, primarily pressure and volume, of the adjacent or connecting compartments or chambers. Ventricular and atrial contraction are the drivers that advances the flow of blood through the circuit; time-varying elastance functions (Sagawa et al., 1973; Defares et al., 1963) are most commonly implemented to recreate ventricular and atrial contraction. As the model becomes more advanced, additional components and functions that account for heart chamber interactions, pericardial influence, and neuro-auto-regulatory mechanisms, such as the baroreflex, and the interaction with the respiratory system may be included.

### 2.3 Current approaches to modelling heart valves and valvular disease

Accurate modelling of heart valves and valvular diseases is crucial for understanding and predicting the heart’s haemodynamic behavior. Approaches to modelling heart valves is varied in the literature and will largely depend on the intended purpose of the cardiovascular model. This section describes the current reduced-dimensional approaches in modelling heart valves and valvulopathy, focusing on common mathematical representations to describe pressure-driven flow.

The most common mathematical representation of a heart valve is by a unidirectional flow function, commonly expressed as a diode connected to a compliance element. Forward flow occurs as a function of the pressure differential between the two adjoining compartments and a resistance value assigned to the valve. In the open state, a normal healthy valve imposes minimal or no resistance to forward transvalvular flow, so parameters for resistance are typically very low, for example, 0.01 mmHg/L/minute (Frank and Hoppensteadt, 2002), or may be absent altogether. The diode representation of the heart valve is one of an ideal valve, where pressure differential mediated opening and closure of the valve is instantaneous. Thus, the valve exists in only two states, fully open or fully closed. As a consequence, pressure, and flow wave-form morphologies will often contain abrupt or non-smooth transitions, particularly at the point of valve opening and closure.

Valve models that are created based on the principle of the conservation of mass and energy, allow for the simulation of non-linear resistance, and provide an accurate portrayal of the relationship between orifice area and pressure gradient. In a 1985 study, Donovan modeled the aortic valve with non-linear resistance, in which flow through the valve was a function of the density of the fluid, an ascribed discharge coefficient, the diameter of the opening orifice, and the volumetric flow rate (Donovan and Sauer, 1985). In the past 20 years, advancements have been made in modelling the dynamic nature of valve opening and closure. A model incorporating a dynamic mitral valve opening area was developed by Szabó et al. (Szabo et al., 2004). In their study, a linear second-order differential equation described valve area opening as a function of changes to pressure and flow where viscous and inertial forces are represented as constants. The authors demonstrated physiologically accurate transmitral flow waveforms for a normal valve and a mechanical prosthetic valve. Korakianitis and Shi described a dynamic valve model based on differential equations incorporating the effect of pressure, flow, and friction on the opening and closing of valve leaflets (Korakianitis and Shi, 2006a; Korakianitis and Shi, 2006b). Valve orifice area is determined by leaflet angular displacement, represented by a cosine function, which is dependent on the pressure exerted on the leaflets. The frictional effects imposed by the leaflet on blood flow are assumed to be proportional to the leaflet’s angular velocity. Herein, the modelling of valvular stenosis can be performed by limiting the maximal opening angle of the leaflets. Likewise, regurgitation can be modeled by increasing the minimum valve closing angle, thereby leaving clearance for flow reversal during diastole (Korakianitis and Shi, 2006a). However, a study that used this approach to simulate valvulopathy according to disease grading (Scarsoglio et al., 2016), produced haemodynamics measures that varied in their agreement with the severity ranges indicated by clinical guidelines. In aortic stenosis (AS), the transvalvular mean pressure gradient is a standard measure of grading severity, primarily determined by the aortic valve area and transvalvular flow rate. Typically, a mean pressure gradient of less than 20 mmHg indicates mild AS, 20–40 mmHg indicates moderate AS, and greater than 40 mmHg indicates severe AS (Baumgartner et al., 2009). In this study, increasing AS severity was achieved through creating valve areas of 2.0 cm^2^ (mild), 1.2 cm^2^ (moderate), and 0.9 cm^2^ (severe), which yielded mean pressure gradient gradients of 26, 54, and 87 mmHg, respectively. These values are higher than what would be expected clinically for these valve areas. Conversely, the simulation of mitral stenosis (MS) produced MPGs that aligned well with clinical guidelines. However, in the simulation of mitral regurgitation (MR), modeled regurgitant orifice areas for mild and moderate MR resulted in regurgitant volumes of 34 and 64 mL, respectively, which corresponds to moderate and severe MR according to clinical guidelines (Lancellotti et al., 2010).

Recently, the simulation of valvulopathy has been performed using an object-oriented modelling approach (Šeman et al., 2023). Here, the modelling of physical systems in an *in silico* environment is achieved using equation-based elements and mathematical operations that represent a physical component (The MathWorks, 2022). These components are usually presented as a graphical or textual icon and can be connected to each other to form the system under design (Kulhánek et al., 2014). Modelling-based environments such as Modelica (Ježek et al., 2017; Moza et al., 2017) and Simscape-Matlab (de Canete et al., 2013; King et al., 2018; Rosalia et al., 2021; King et al., 2019) have been previously used to model a closed-loop cardiovascular system. In the Simscape environment, component valves comprise of mathematical equations based on the continuity of mass and energy that describe valve function and the pressure and flow dynamics of fluid through the valve (King et al., 2019; The MathWorks Inc, 2020). Within these environments, a vast range of physical properties of components can be specified, including the dimensions of compliant pipes, opening and regurgitant orifice areas for valves, and fluid viscosity and density (The MathWorks Inc, 2020).

## 3 Modelling-based research in valvular heart disease

Computational modelling has enormous potential to significantly advance the understanding, diagnosis, and treatment of VHD. A key strength of cardiovascular modelling research is the ability to investigate specific conditions with great controllability and reproducibility, including the acquisition of precise data for a wide variety of parameters. This allows for tailored simulation of clinical scenarios and precise quantitation of the effects of variables of interest.

This section of the review showcases studies that utilized lumped-parameter computational models of the cardiovascular system to unveil clinical insights into VHD. High-dimensional modelling studies, including structural finite element analysis, computational fluid dynamics, and fluid–structure interaction, will not be discussed and have been previously outlined elsewhere (Toma et al., 2022). A summary of key modelling-based VHD studies is presented in Table 2.

**TABLE 2:** Key themes in modelling-based valvular heart disease research.

Theme	Study focus
Haemodynamic consequences of valvulopathy	• Haemodynamic effect of altered loading conditions in AS (Garcia et al., 2006; Keshavarz-Motamed et al., 2014; Popovic et al., 2005; Ben-Assa et al., 2019) and MR (Šeman et al., 2023; Inuzuka et al., 2016)
	• Characterization of transvalvular flow and pressure changes in AS (Šeman et al., 2023; Garcia et al., 2006; Keshavarz-Motamed et al., 2014; Ben-Assa et al., 2019; Syomin et al., 2019; Laubscher et al., 2022), AR (Syomin et al., 2019), MS (Syomin et al., 2019), MR (Šeman et al., 2023; Syomin et al., 2019), TR (Hemalatha et al., 2010) and PR (Hemalatha et al., 2010)
	• Haemodynamic changes associated with acute and chronic MR (Ripoli et al., 1998)
	• Evaluation of left ventricular remodelling in response to AS, AR, and MR (Maksuti et al., 1985)
	• Mechanism of impaired coronary flow reserve in AS (Garcia et al., 1985)
	• Arrhythmogenic effect of AS,AR,MS and MR (Pearce and Kim, 2022)
Interaction with co-existing cardiovascular pathology	• Haemodynamics of AS (Šeman et al., 2023; Popovic et al., 2005), and MR (Šeman et al., 2023; Inuzuka et al., 2016) with co-existing heart failure
	• Impact of concomitant MR on AS (Šeman et al., 2023)
	• Haemodynamic impact of AR, AS, MR, MS in the setting of atrial fibrillation (Scarsoglio et al., 2016)
	• Effect of atrioventricular valve regurgitation in congenital single ventricle (Pant et al., 2018; Schiavazzi et al., 2017)
Patient-specific clinical prediction	• Patient-specific diagnostic and clinical prediction tool for quantifying cardiovascular haemodynamics and key heart function metrics (Keshavarz-Motamed, 2020; Baiocchi et al., 2021)
	• Patient-specific modelling of stroke work pre- and post-transcatheter aortic valve replacement and its correlation to quality-of-life (Ben-Assa et al., 2019)
	• Patient-specific modelling of coronary artery haemodynamics pre- and post-transcatheter aortic valve replacement (Garber et al., 2023)
	• Prediction of invasive haemodynamic metrics in a patient with congenital single ventricle and atrioventricular valve regurgitation (Schiavazzi et al., 2017)
Diagnostic advancements	• Validation of novel index, normalized stroke work, to assess the haemodynamic load imposed on the left ventricle in patients with AS (Keshavarz-Motamed et al., 2014)
	• Role of myocardial performance index in the setting of MR under different loading conditions (Inuzuka et al., 2016)
	• The use of left ventricular stroke work and vascular impedance as metrics to improve the characterization of patients with AS (Ben-Assa et al., 2019)
	• Value of assessing pulmonary vein flow in the echocardiographic assessment of MR severity (Grimes et al., 1995)
	• Quantitation of the impact of MR on haemodynamic indices of AS severity (Šeman et al., 2023)
Simulation of therapeutic strategies	• Aortic valve bypass for the treatment of severe AS (Benevento et al., 2015)
	• The haemodynamic benefit of gradual versus abrupt correction of MR and TR (Walmsley et al., 2019)
	• Effects of sodium nitroprusside in aortic stenosis associated with severe heart failure (Popovic et al., 2005)
	• Optimal timing of correction of atrioventricular valve regurgitation during staged reconstructive surgery of congenital single ventricle (Pant et al., 2018)
	• Effectiveness of pulsatile and continuous LVAD therapy in the setting of AR or MR (Kim et al., 2016)
	• Impact of TR on the haemodynamic effects of RVAD treatment (Punnoose et al., 2012)
	• Haemodynamic Impact of venoarterial extra corporeal membrane oxygenation in the setting of AR (Zhu et al., 2024)

Abbreviations: AR, aortic regurgitation; AS, aortic stenosis; MR, mitral regurgitation; MS, mitral stenosis; PR, pulmonary regurgitation; TR, tricuspid regurgitation; RVAD, right ventricular assist device; LVAD, left ventricular assist device.

### 3.1 Haemodynamic insights into the progression of valvular heart disease

VHD is inherently progressive in nature. Valvular stenosis or regurgitation disrupts normal haemodynamics, resulting in increased cardiac pressures and workload. Over time, this can lead to ventricular dysfunction, and the development of symptoms such as shortness of breath, fatigue, chest pain, and syncope. These symptoms significantly impact quality of life and often indicate the necessity for medical or surgical intervention for the alleviation of symptoms and prevention of further deterioration.

Understanding the variables that underpin disease progression is crucial for assessing the severity and potential outcomes of VHD. In silico experimentation using cardiovascular models allows for the investigation of VHD under highly controlled and repeatable conditions. With this approach, the continuum of valvular stenosis and regurgitation can be simulated, and a wide array of haemodynamics can be measured with high precision.

In a study by Garcia and colleagues (Garcia et al., 2006), AS with increasing severity was simulated using a LPM, and the relationship between aortic valve area and left ventricular (LV) stroke work was quantified. In their study, LV stroke work was shown to remain relatively stable in the ranges of mild and moderately severe AS. As the aortic valve area reduced below <1.0 cm^2^, small decreases in effective orifice area induced drastic increases in LV stroke work. Indeed, this orifice area corresponds to the approximate turning point in which patients experience the onset of symptoms and mortality risk rises significantly (Mehrotra et al., 2018). In the same study, the authors showed increasing grades of hypertension resulted in a quasi-linear increase in LV stroke work (Garcia et al., 2006). As such, hypertension was highlighted as a potentially important contributor to the discordance commonly seen between patients’ imaging derived AS severity, their onset of symptoms, and the extent of symptoms.

The presence of impaired coronary flow reserve in patients with severe AS is associated with an increased risk of myocardial ischemia symptoms, LV dysfunction, and sudden death. (Michail et al., 2018). To investigate the complex interplay between coronary flow reserve and AS, a sophisticated LPM of the cardiovascular system that incorporated coronary inflow was employed to simulate progressive AS (Garcia et al., 1985). Simulated coronary flow waveforms were found to be comparable to those reported in the literature for healthy valves and AS. The results revealed a discernible correlation between the progressive AS severity and a decrease in coronary flow reserve. At valve areas of 1.0 cm^2^ and below coronary flow reserve decreased markedly. For example, compared to no AS with a valve area of 4.0 cm^2^, valve areas of 1.0 cm^2^ and 0.5 cm^2^ were associated with approximate 25 and 50 percent reductions in coronary flow reserve, respectively. The reductions in coronary flow reserve were explained in large part by i) a decrease in coronary perfusion pressure leading to reduced myocardial supply, ii) decreased diastolic duration, and iii) increased myocardial metabolic demand associated with increased LV workload. Given the adverse outcomes associated with a very low coronary flow reserve, the authors expressed that their findings provide some support in favor of raising the level of indication for aortic valve replacement in asymptomatic cases of very severe AS (V_max_ ≥ 5 m/s or mean transaortic pressure gradient ≥60 mmHg), which at the time of publication was a class 2b indication (Bonow et al., 2008), and has since increased to a class 2a indication (Otto et al., 2020).

### 3.2 Impact of co-existing cardiovascular disease

In patients with VHD, the presence of co-existing cardiac diseases, such as heart failure or atrial fibrillation are frequently found. Despite extensive study, pathophysiologic interactions between valvulopathy and co-existing cardiac disease remain incompletely defined. Modelling-based studies enable the investigation of the contributory role of additional cardiac pathologies in the continuum of specific valvulopathy, and vice versa, thus facilitating a more comprehensive understanding of the complex interplay of the key factors at play.

**FIGURE 5:**
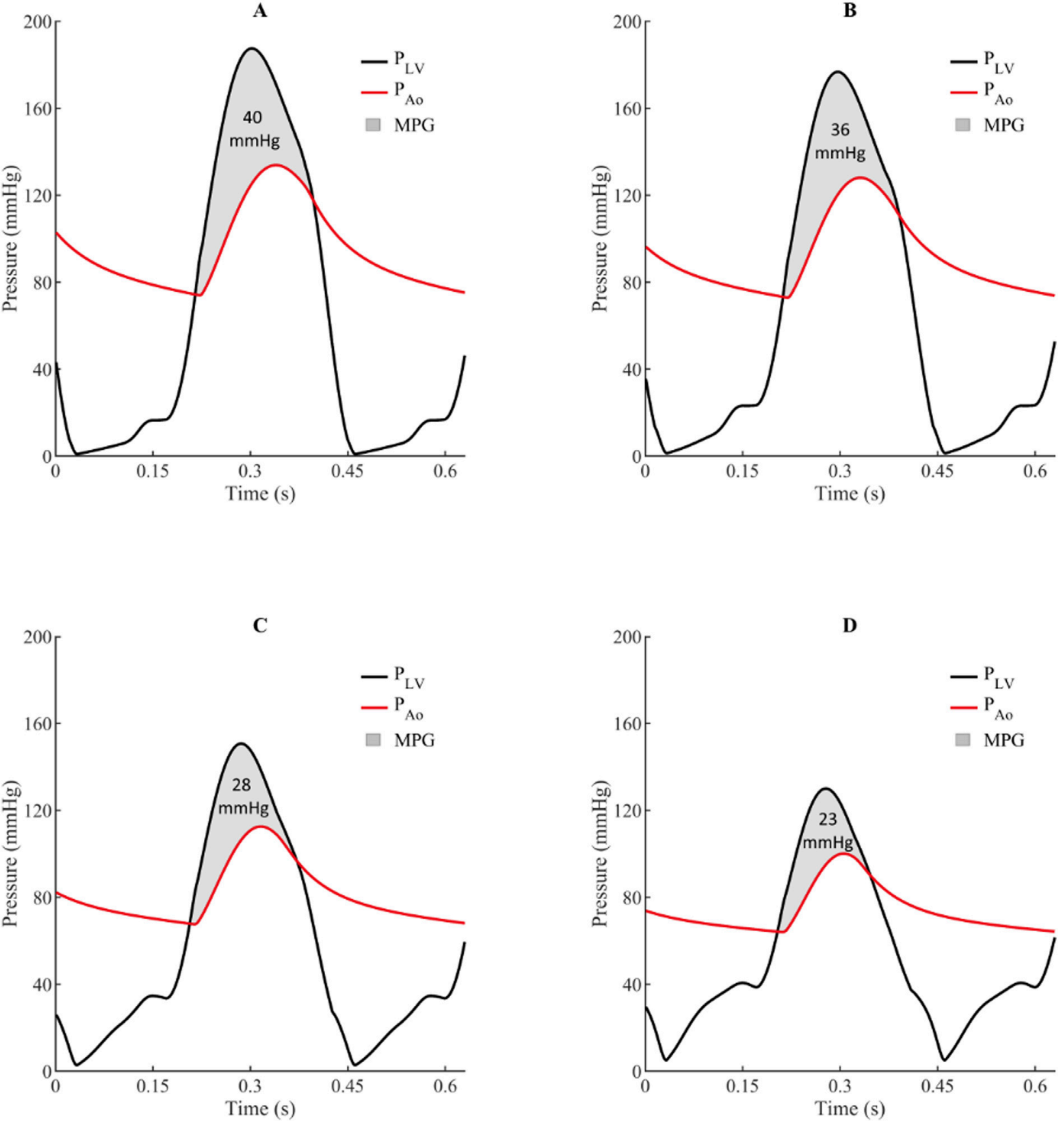
Simulated impact of concomitant mitral regurgitation on left ventricle and aortic pressures waves and mean pressure gradient for aortic stenosis (aortic valve area = 1.0 cm^2^).^21^ **(A)** = Isolated aortic stenosis, **(B)** = Aortic stenosis with mild mitral regurgitation, **(C)** = Aortic stenosis with moderate mitral regurgitation, and **(D)** = Aortic stenosis with severe mitral regurgitation. MPG indicates transaortic mean pressure gradient; P_Ao,_ aortic pressure; and P_LV_, left ventricular pressure.

Mixed and multiple valvular diseases are also a frequent occurrence in clinical practice, however, there are limited studies investigating the adverse haemodynamics of such states and its implications on clinical outcomes. The impact of concomitant MR on AS haemodynamics was investigated using a LPM and echocardiographic data from a large cohort of patients with AS (Šeman et al., 2023). Figure 5 shows simulated LV and aortic pressure waveforms and for progressively worsening MR (mild, moderate, and severe) in the setting of severe AS with an aortic valve area 1.0 cm^2^. For severe AS, simulations showed that compared to isolated AS, the presence of mild, moderate, and severe MR was associated with transaortic mean pressure gradient reductions of 10%, 29%, and 40%, respectively. These results when then used to calculate an *adjusted* mean pressure gradient in a cohort of 1427 patients with severe AS, based on MR severity. The authors found that co-existing MR, even if mild, was a significant contributor to echocardiographic discordance between aortic valve area and mean pressure gradient. And of patients with low-gradient AS and concomitant MR, half would reclassify as high-gradient AS based on their adjusted-mean pressure gradient. The authors highlighted the risk of underestimating AS severity in patient with concomitant MR and discussed the potential benefits of an *adjusted* mean pressure gradient, based on careful MR quantitation. This approach could be especially important for patients demonstrating echocardiographic discordance or borderline severe AS, as it may provide a more accurate assessment of the true AS severity in the presence of confounding MR.

### 3.3 Improved diagnostic evaluation

**FIGURE 6:**
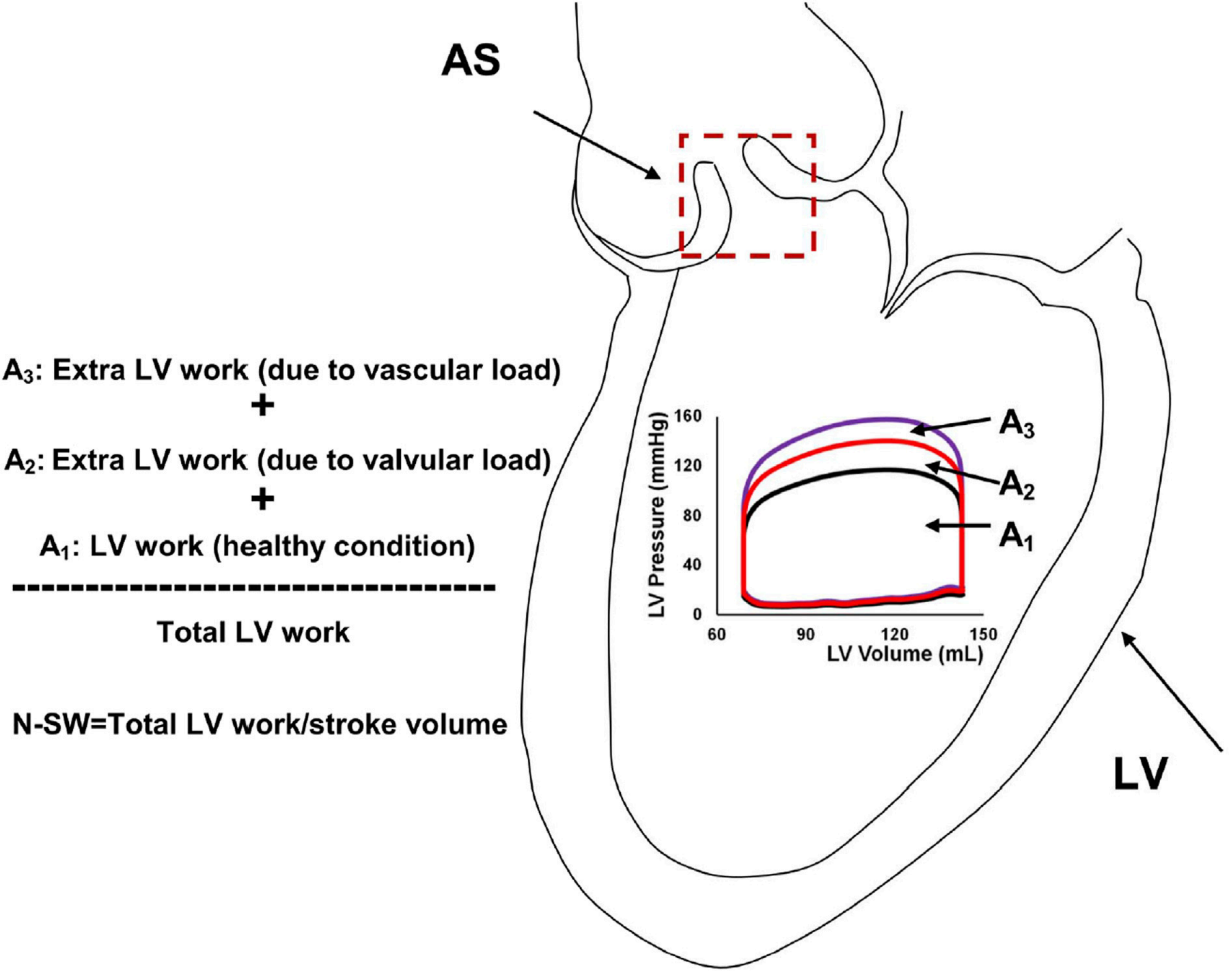
Schematic of left ventricular stroke work and normalized-stroke work in aortic stenosis. AS, aortic stenosis, LV, left ventricle; N-SW, normalized-stroke work. (Figure taken from Keshavarz-Motamed et al. (2014)).

Mathematical modelling has recently made inroads as tool for optimizing the diagnostic evaluation of VHD. An example of such a study is the paper by Keshavarz-Motamed et al., which introduced a new metric for evaluating AS severity ‘normalized stroke work’ (Keshavarz-Motamed et al., 2014), This index is calculated by dividing the estimated LV stroke work by the stroke volume and represents the global haemodynamic load faced by the LV to eject a unit volume of blood (see Figure 6). In the study, a LPM was informed by patient-specific data from cardiac imaging to estimate their normalized stroke work. The authors found normalized stroke work to be highly correlative to effective orifice area and largely independent from variations in flow rate. The authors highlighted the potential diagnostic and prognostic value of this novel index if added to current standard indices of AS severity, for the evaluation of low-flow AS states, where the determination of severity is challenging.

Inuzuka and colleagues modeled the effect of heart failure and MR on the myocardial performance index (MPI) (Inuzuka et al., 2016). Also known as the Tei index, the MPI is a doppler-derived measure for global ventricular function which is calculated using isovolumic and ejection times. In the simulated progression of heart failure and MR, the authors showed paradoxical improvements in the MPI with worsening disease. The combination of volume overload, reduced arterial compliance, and restricted LV filling, led to a marked overestimation of LV performance, despite reduced LV contractility and elevated end-diastolic pressure. The authors highlighted due consideration of these haemodynamic confounders when using the MPI as a measure of cardiac function in clinical assessment.

### 3.4 Education and training

Over the last 2 decades, there has been a gradual emergence of cardiovascular modelling tools for educational purposes. These models, which have strong foundations in demonstrating normal physiological processes to users, have evolved to simulate a variety of different cardiac pathologies, including VHD. Cardiovascular models such as Harvi-Online (Burkhoff and Schleicher), CircAdapt (Arts et al., 2005), and CVSim (Heldt et al., 2010) have emerged that allow users to simulate a variety of different cardiac pathologies, including VHD, and visualize an array of cardiovascular haemodynamics in real time. These educational tools provide an interactive platform for learning the key principles of cardiovascular physiology and pathophysiology, which may be limited to acquire in other media, such as textbooks. For students, educators, and clinicians to take full advantage of these tools in the future, it is important to make them easily accessible and user-friendly.

### 3.5 Pre-clinical investigation and hypothesis generation

Cardiovascular modelling has been used to study the complex behavior of the cardiovascular system under different conditions and interventions, which would otherwise be very challenging or prohibitive to perform on animals or humans. Additionally, modelling can help clinical researchers design more efficient and effective experimental protocols, and to identify the most crucial variables to investigate. The role of novel surgical techniques and exploring strategies for optimally delivering mechanical circulatory support to patients with VHD are just some of the areas being explored with modelling approaches.

**FIGURE 7:**
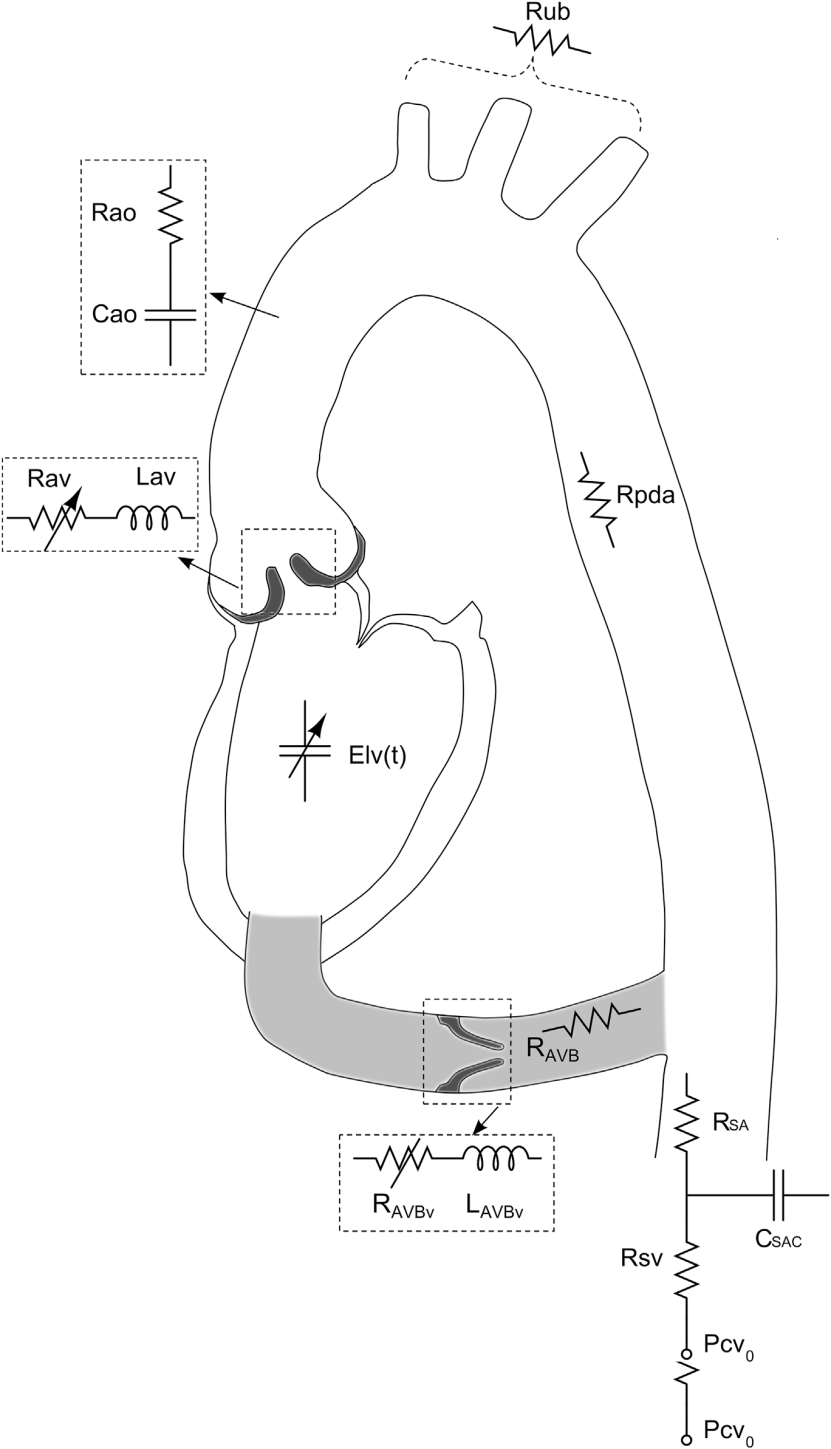
Schematic representation of the lumped parameter model used to simulate left-sided heart in presence of aortic stenosis and/or left ventricular-aortic conduit. (Figure adapted from Benevento et al. (2015)).

For patients with severe AS in whom valve replacement is contraindicated, the use of aortic valve bypass is previously proposed surgical procedure surgical procedure for the relief of left ventricular outflow tract obstruction (Gammie et al., 2008). An effective double outlet ventricle is created, whereby the LV outflow conduit is connected to the descending aorta (Figure 7). In simulations of severe AS, aortic valve bypass resulted in significant reductions in LV stroke work, transvalvular pressure gradient, and LV end-diastolic pressures (Benevento et al., 2015). These results indicate aortic valve bypass to be a potentially viable solution for patients with severe AS and who are contraindicated to both surgical or transcatheter aortic valve replacement.

Deterioration of LV function post mitral valve surgery for chronic MR is a common complication that portends worse clinical outcomes (Quintana et al., 2014). Walmsley et al. hypothesized that the abrupt change to ventricular loading conditions following valve replacement is a key contributor to postoperative ventricular dysfunction (Walmsley et al., 2019). To investigate this hypothesis, the authors used a cardiovascular model to compare the effects of abrupt and gradual correction of mitral and tricuspid regurgitation on cardiac function. Results showed that simulated surgical correction of tricuspid regurgitation (TR) and MR resulted in a sudden spike of ventricular stress that could exacerbate post-operative myocardial depression. On the other hand, no spike was observed in the simulation of gradual correction of TR and MR. The authors proposed a potential future role for strategies that gradually correct valve regurgitation, to reduce ventricular overload and facilitate gradual reverse remodelling.

The impact of aortic and mitral valve regurgitation on the pumping efficacy of a left ventricular assist device (VAD) with the pulsatile or continuous flow was the subject of a simulation study by Kim and colleagues (Kim et al., 2016). The authors found that pulsatile VAD treatment delivered the highest pumping efficacy in cases of MR compared to continuous flow, owing to a greater overall afterload reduction achieved through pulsatile treatment. Additionally, even with the development of MR during VAD treatment, mean arterial blood pressure and cardiac output remained constant, regardless of the MR severity. Meanwhile, for severe AR, improvements in cardiac output and mechanical load reduction were only marginal for both pulsatile and continuous LVAD treatment. In a similar study, potential implications of TR on the efficacy of a right-sided VAD was investigated (Punnoose et al., 2012). The authors showed the presence of TR did not significantly affect VAD performance, nor did the VAD significantly impact the severity of TR. Interestingly, the authors noted that in the case of severe pulmonary hypertension and where the VAD draws blood from the right atrium, significant TR may be advantageous; this configuration increases the potential for the right ventricle to retain some native pulsatility, reducing flow stagnation and clot development.

### 3.6 Precision medicine and patient-specific prediction

**FIGURE 8:**
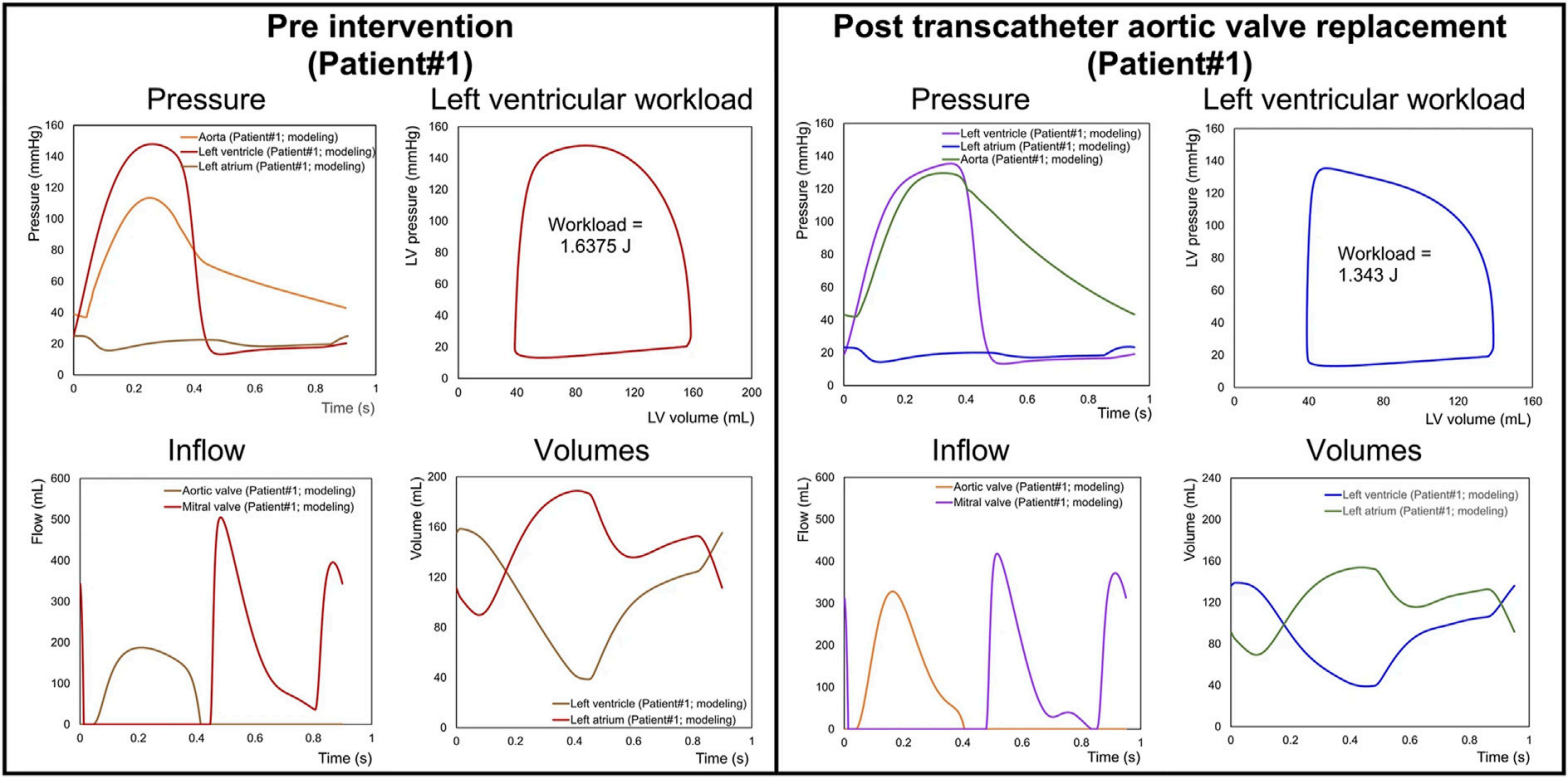
Predicted haemodynamics in a C3VI patient from baseline to 90 days post-transcatheter aortic valve replacement (TAVR). Pre-TAVR: severe aortic stenosis (effective orifice area (EOA) = 0.5 cm^2^); mild aortic regurgitation (AR); moderate to severe mitral regurgitation (MR); moderate to severe concentric hypertrophy; LV ejection fraction, 50%; arterial pressures, 115/40 mmHg; forward LV stroke volume, 54 mL. Post-TAVR: aortic valve (EOA = 1.6 cm^2^); mild to moderate AR; moderate to severe MR; hypertension, moderate to severe concentric hypertrophy; LV ejection fraction, 60%, arterial pressures, 140/45 mmHg; forward LV stroke volume, 53 mL. (Figure adapted from Keshavarz-Motamed (2020)).

There is a growing interest in developing patient-specific models of cardiovascular disease that can be used to improve diagnosis and tailor treatment strategies for individuals. Already, such clinical tools are beginning to emerge (Keshavarz-Motamed et al., 2014; Keshavarz-Motamed, 2020). Keshavarz-Motamed adapted a previously studied lumped parameter model for the basis for an innovative imaged-based patient-specific diagnostic, monitoring, and predictive tool (called C3VI-CMF) (Keshavarz-Motamed, 2020). The model included valvular updates to facilitate additional valve pathologies, including mitral valve disease, thus allowing for the simulation of mixed valvular disease. The C3VI-CMF tool was validated against cardiac catheterization data from a cohort of patients with complex valvular disease. The tool uses a selection of key parameters obtained from the patient’s echocardiogram to inform the model, which generates accurate predictions of heart function metrics, such as for patients pre- and post-valvular intervention. Figure 8 illustrates the use of the C3VI in a patient pre and post transcatheter aortic valve replacement (TAVR). In the future, such precision medicine tools could allow for simulating the patient-specific effects of different combinations of drug therapies, the timing and type of surgical strategy, and the response to mechanical circulatory support. Additionally, in patients with early or late disease, predictions for long-term progression without treatment could be predicted.

## 4 Limitations of modelling-based research of valvular heart disease

Mathematical models are simplified representations of complex processes, and as such, there are inherent limitations in their attempt to capture all the complexities of reality. Models of the cardiovascular system and cardiac pathology are limited by the experimental or clinical data, which are used to parameterize the equations that describe the processes being studied. Models are typically developed with parameters informed by literature data, population averages, and animal studies, to first create a healthy generic cardiovascular state (Garber et al., 2021). For the simulation of cardiac pathology, the model is then adapted and calibrated based on obtainable data, which is often limited. As an example, estimating the compliances and resistances in vessels is challenging in both clinical settings and animal experiments (Shimizu et al., 2018). This challenge is only compounded by the need to estimate these values for specific cardiac disease states. As a result, it is often necessary to make certain assumptions about these values at the beginning of the simulation, and then calibrate them to physiologically appropriate values for the disease state modeled. Typically, an archetypal representation of a specific disease or physiological state is sought. In this regard, the model is not meant to capture the vastness of individual variations in normal physiology nor the heterogeneity of pathological states. Despite this, patient-specific modelling approaches have been developed, in which a patient’s individual clinical and imaging data are used to inform model parameterization and patient-specific simulation results are generated (Keshavarz-Motamed, 2020; Garber et al., 2021).

Every modelling-based study has its own specific limitations, which are often closely related to the validity of the model being used. Validity is defined as the extent to which a model fulfils the intended objectives for which it was formulated (Leaning et al., 2016). Thus, the impact of any model deficiencies, on the validity of the model and the simulation results are dependent on the model’s intended purpose and context of use (Pathmanathan and Gray, 2018). Among modeling-based VHD studies reviewed, the approaches to validating specific valvulopathy have varied. These include qualitative or quantitative comparisons of pressure or flow waveforms from clinical or literature data (Keshavarz-Motamed et al., 2014; Benevento et al., 2015; Hemalatha et al., 2010), or by comparing to key echocardiographic indices for a specific valvulopathy according to severity ranges outlined in clinical guidelines (Šeman et al., 2023; Punnoose et al., 2012; Mynard et al., 2012; Fresiello et al., 2015). The methods for cardiovascular model validation differ significantly and have been discussed in detail elsewhere (Pathmanathan and Gray, 2018; Leaning et al., 2016).

## 5 Future directions

As computational cardiovascular modelling continues to advance in tandem with increasing computational capabilities, simulation-based tools are set to become indispensable in both research and clinical practice. To fully harness their potential, it is imperative to foster interdisciplinary collaboration, thereby enhancing the availability and accessibility of these tools.

Key areas in cardiovascular modelling that are poised to make significant advancements are shown in Table 3. These advancements, driven by technological innovations and interdisciplinary research, promise to significantly improve our ability to model complex disease phenotypes and make patient-specific predictions. By integrating computational modelling with other research methodologies—such as *in vitro* experiments, *in vivo* studies, and clinical data—researchers can achieve a more comprehensive and nuanced understanding of disease progression. This multifaceted approach is crucial for unveiling mechanistic insights and developing effective personalized or precision medicine strategies.

**TABLE 3:** Research directions in computational modelling of valvular heart disease.

Themes	Key Directions
Combining resaearch methods	• Artificial intelligence and machine learning
	• *In-vitro*, *in-vivo*, and clinical study design optimization
	• Additional data analysis and interpretation through simulation
	• Linking models with clinical outcome data
	• Streamlined processes for imaging and haemodynamic data acquisition
Pre-clinical research	• Testbed for new therapies and interventional strategies
	• Optimizing existing treatment approaches
	• Device development
	• Hypothesis generation
Diagnostic advancements	• Developing new metrics for risk and severity assessment
	• Improved quantitation of key haemodynamics
	• Imputing missing or unobtainable measures
	• Integration with clinical, diagnostic, and imaging software
	• Reconciling clinical and imaging discordance
Multiscale models	• Cellular metabolism and tissue function
	• Electrophysiology and arrhythmias
	• Respiration and gas exchange
	• Cardiac remodelling
	• Multiple and mixed cardiac pathologies
	• Non-cardiac disease, e.g., chronic lung disease
Education and training	• Physiology and medical students
	• Cardiac imaging training
	• Clinical study design
	• Haemodynamic simulator for clinical simulation training
	• Integration with clinical simulation trainers, e.g., mannequins
Access and usability	• Improved user interfaces for use by non-experts
	• Accessible stand-alone software packages or online-apps
	• Instructional and trouble-shooting resources
	• Tailored tools for clinicians, students, educators, and researchers
	• Streamlined data integration
Precision medicine and patient-specific modelling	• Integration of data from clinical records, imaging, and diagnostic databases
	• Personalized clinical prediction and assessment
	• Treatment planning and optimization
	• Mechanical circulatory support control

The progression of cardiovascular disease is complex and involves interactions at multiple scales, from molecular and cellular processes to organ system interactions. Multiscale modelling involves integrating multiple different mathematical models allowing for the representation of multiple different phenomena across different measurable domains of time, space, and/or function (Walpole et al., 2013). Currently, there is a vast and promising scope for the expansion of multiscale models of VHD. At the cellular and tissue level, multiscale cardiovascular models allow for the simulation of the chronic cardiac remodelling changes that occur in response to worsening valvulopathy (Arts et al., 2005; Li et al., 1997; Maksuti et al., 1985). Significant progress has already been made to couple complex cardiac electrophysiological models with a lumped-parameter model for closed-loop cardiovascular simulation (Heldt et al., 2010; Regazzoni et al., 2022). This coupling forms the foundation for more comprehensive investigations into arrhythmias, conduction disorders, and pacing therapies. Indeed, multiscale models have value in the simulation of multiple and mixed disease states, where progression is driven by multiple interdependent factors.

There have been increasing efforts in clinical research to better examine the clinical heterogeneity within patient disease groups and identify subgroups of patients who share similar characteristics. For clinicians assessing patients with multiple cardiac pathologies, it is inherently difficult to discern the relative pathological consequences of a valvulopathy, over an arrhythmia, such as atrial fibrillation, and/or heart failure with reduced or preserved ejection fraction (Scarsoglio et al., 2016). This has implications for monitoring disease progression or response to therapy to any one pathology, in particular for patients deemed to be borderline indicated for valve replacement strategies. Indeed, it is not uncommon that such co-morbid patients to fail to derive symptomatic or mortality benefits from valvular intervention (Puri et al., 2016). The lack of benefit in these patients highlights the need for more personalized approaches that consider the interplay of multiple disease processes. Modelling provides the opportunity to quantify the contributory effect of coexisting diseases (both cardiac and non-cardiac) on symptom manifestation, disease progression, and response to therapy. Translated to the patient’s bedside, such knowledge can allow clinicians to make more informed decisions on treatment strategies and prognosis.

## 6 Conclusion

Computational modelling of the cardiovascular system has emerged as a powerful tool that allows detailed examination into the pathophysiology of VHD allowing for consolidation of existing knowledge and highlighting of knowledge gaps. Models facilitate hypothesis generation and allow for the exploration of novel ideas in the pre-clinical context. More recently, advancements have been made to link models to patient-specific data, to gain mechanistic insights and clinical predictions tailored to an individual patient. Ultimately, such patient-specific modelling approaches could be used in the clinical setting as an adjunct for patient diagnostic evaluation, prognostication, and the tailoring of treatment strategies. To date, the contribution of VHD modelling research has been predominantly in the realm of basic science. Opportunities for clinical translation are vast and are likely to be realized in the near future.

## Abbreviations

AS, Aortic stenosis; AR, Aortic regurgitation; LPM, Lumped parameter model; LV, Left ventricle; MR, Mitral regurgitation; MS, Mitral stenosis; PR, Pulmonary regurgitation; TR, Tricuspid regurgitation; VAD, Ventricular assist device; VHD, Valvular heart disease.
